# Psychotropic drugs up-regulate the expression of cholesterol transport proteins including ApoE in cultured human CNS- and liver cells

**DOI:** 10.1186/1471-2210-9-10

**Published:** 2009-08-29

**Authors:** Audun O Vik-Mo, Johan Fernø, Silje Skrede, Vidar M Steen

**Affiliations:** 1 Dr. Einar Martens' Research Group for Biological Psychiatry and Bergen Mental Health Research Center, Department of Clinical Medicine, University of Bergen, Norway; 2 Center for Medical Genetics and Molecular Medicine, Haukeland University Hospital, Helse Bergen HF, Norway

## Abstract

**Background:**

Disturbances in lipid homeostasis and myelination have been proposed in the pathophysiology of schizophrenia and bipolar disorder. We have previously shown that several antipsychotic and antidepressant drugs increase lipid biosynthesis through activation of the Sterol Regulatory Element-Binding Protein (SREBP) transcription factors, which control the expression of numerous genes involved in fatty acid and cholesterol biosynthesis. The aim of the present proof-of-principle study was to investigate whether such drugs also affect lipid transport and export pathways in cultured human CNS and liver cells.

**Results:**

Quantitative PCR and immunoblotting were used to determine the level of lipid transport genes in human glioblastoma (GaMg) exposed to clozapine, olanzapine, haloperidol or imipramine. The effect of some of these drugs was also investigated in human astrocytoma (CCF-STTG1), neuroblastoma (SH-SY5Y) and hepatocellular carcinoma (HepG2) cells. We found significant transcriptional changes of cholesterol transport genes (*ApoE, ABCA1, NPC1, NPC2, NPC1L1*), which are predominantly controlled by the Liver X receptor (LXR) transcription factor. The up-regulation was observed after 24 to 48 hours of drug exposure, which is markedly delayed as compared to the drug-induced SREBP-controlled stimulation of lipid biosynthesis seen after 6 hours.

**Conclusion:**

Our data show that stimulation of cellular lipid biosynthesis by amphiphilic psychotropic drugs is followed by a transcriptional activation of cholesterol transport and efflux pathways. Such effects may be relevant for both therapeutic effects and metabolic adverse effects of psychotropic drugs.

## Background

Antipsychotic and antidepressant drugs are imperative in the treatment of schizophrenia and affective disorders. These drugs exert their therapeutic effects at least in part through perturbation of the dopamine-, noradrenaline- and serotonin neurotransmitter systems in the brain, but additional molecular mechanisms of action are likely to contribute to their clinical effect. We have demonstrated that several antipsychotics and antidepressants increase lipid biosynthesis in cultured human CNS cells [1-4]. This drug-induced stimulation of cellular lipogenesis could represent a novel mechanism of psychotropic drug action in the brain, since glia-produced lipids, including cholesterol, play important roles in myelination and synaptogenesis [5,6]. Interestingly, several studies have indicated disrupted glial function, as well as lipid and myelin abnormalities, in schizophrenia and affective disorders [7-11]. The drug-mediated lipogenic effect could also be relevant for the associated serious metabolic adverse effects, such as weight gain and dyslipidemia. Indeed, some of the psychotropic drugs increase the expression of lipid biosynthesis genes in cultured hepatocytes and adipocytes [1,3,12-14], as well as in blood cells from olanzapine-treated patients [15].

The increased lipid biosynthesis is mediated through activation of the sterol regulatory element-binding protein (SREBP) transcription factors, which control the expression of genes involved in cellular production of cholesterol (e.g., *3-hydroxy-3-methylglutaryl-CoA reductase; HMGCR*) and fatty acids (e.g., *fatty acid synthase; FASN *and *stearoyl CoA-desaturase; SCD*). The SREBP system is sensitive to cationic amphiphilic drugs, such as antipsychotic and tricyclic antidepressant, through their ability to partly mimic the effects of oxysterols [16].

A prolonged drug-induced stimulation of cellular lipogenesis would be expected to activate intracellular lipid transport and export pathways. The Liver X Receptor (LXR) transcription factor functions as an intracellular oxysterol sensor and controls the expression of central cholesterol transport proteins, such as apolipoprotein E (ApoE), ATP-binding cassette transporter A1 (ABCA1) and Niemann-Pick type C (NPC) protein, along with other important factors in the cholesterol homeostasis of the central nervous system and peripheral tissue [17,18].

The aim of this study was to determine if cationic amphiphilic psychotropic drugs known to activate the SREBP-system also have effects on the expression of LXR-related lipid transport and export pathway genes in cultured human CNS and liver cells.

## Results

Human glioblastoma cells (GaMg) were exposed to clozapine (25 μM), olanzapine (10 μM), haloperidol (10 μM) and imipramine (10 μM) for 6, 12, 24 or 48 hours. The choice of drugs and the different drug concentrations was based on their structural similarity and our previous work [1-4], but also taking into consideration the large differences in the clinical dosage of these drugs. As expected, all the drugs induced a substantial early increase in mRNA level of the SREBP-controlled *3-hydroxy-3-methylglutaryl-CoA reductase *(*HMGCR*) gene (Table 1), which is encoding the rate-limiting enzyme in cholesterol biosynthesis. Clearly delayed as compared to the increase in *HMGCR*, we also observed a marked drug-mediated transcriptional activation of the LXR-controlled genes *ApoE, Niemann-Pick type C1 *(*NPC1*) and *Niemann-Pick type C2 *(*NPC2*), reaching maximal values after 24-48 hours of drug exposure with no significant response at 6 hours. *ABCA1 *expression diverged from the other LXR targets, with haloperidol and in part imipramine mediating a significant decrease in the mRNA level, whereas clozapine induced a marked up-regulation at 48 hours (Table 1). We also found that the gene encoding the LXR-isoform dominant in CNS, *LXRβ*, was significantly up-regulated by clozapine while down-regulated by haloperidol, although the effect size was small. *LXRα*, which is a lesser expressed isoform in the CNS, displayed a similar but non-significant trend.

**Table 1:** The effects of psychotropic drugs on the expression of cholesterol biosynthesis and transport genes in cultured human glioma (GaMg) cells.

	HMGCR	APOE	ABCA1	NPC1	NPC2	LXRα	LXRβ
Clozapine (25 μM)							
6 h	2.4 ± 0.1**	1.2 ± 0.1	1.0 ± 0.02	1.1 ± 0.04	1.2 ± 0.1	1.2 ± 0.1	1.2 ± 0.03*
12 h	2.2 ± 0.1**	1.8 ± 0.1**	1.5 ± 0.1	1.1 ± 0.03	1.3 ± 0.01*	1.0 ± 0.1	1.0 ± 0.03
24 h	2.1 ± 0.1**	2.2 ± 0.2**	1.2 ± 0.2	1.6 ± 0.1*	1.9 ± 0.1**	1.5 ± 0.3	1.4 ± 0.02**
48 h	2.2 ± 0.1**	4.5 ± 0.1**	2.6 ± 0.2**	2.5 ± 0.1**	2.6 ± 0.1**	1.1 ± 0.04	1.3 ± 0.02**
Olanzapine (10 μM)							
6 h	1.8 ± 0.3*	1.2 ± 0.1	0.9 ± 0.2	1.2 ± 0.2	0.9 ± 0.3	1.1 ± 0.02	1.2 ± 0.09
12 h	1.3 ± 0.05**	1.1 ± 0.1	0.9 ± 0.1	0.9 ± 0.1	1.0 ± 0.02	0.8 ± 0.03*	1.1 ± 0.06
24 h	1.4 ± 0.06**	1.5 ± 0.4	1.4 ± 0.06**	1.3 ± 0.05**	1.3 ± 0.02**	1.0 ± 0.1	1.3 ± 0.04*
48 h	1.1 ± 0.05*	1.5 ± 0.1*	1.2 ± 0.02**	1.0 ± 0.1	1.2 ± 0.04**	1.1 ± 0.05	1.0 ± 0.04
Haloperidol (10 μM)							
6 h	2.1 ± 0.2**	1.2 ± 0.1	0.6 ± 0.06**	1.2 ± 0.1	1.0 ± 0.1	0.9 ± 0.07	1.1 ± 0.01
12 h	2.1 ± 0.1**	1.5 ± 0.2	0.3 ± 0.03**	1.1 ± 0.02*	1.2 ± 0.05*	0.6 ± 0.07**	0.8 ± 0.07*
24 h	2.2 ± 0.04**	2.3 ± 0.2**	0.3 ± 0.03**	1.7 ± 0.04**	1.8 ± 0.04**	1.1 ± 0.05	1.2 ± 0.3
48 h	1.8 ± 0.01**	2.4 ± 0.2**	0.3 ± 0.04**	1.5 ± 0.07**	1.8 ± 0.06**	1.0 ± 0.03	1.1 ± 0.04
Imipramine (10 μM)							
6 h	2.0 ± 0.1**	1.2 ± 0.1	0.7 ± 0.2*	1.1 ± 0.1	0.8 ± 0.3	1.0 ± 0.07	1.1 ± 0.06
12 h	2.1 ± 0.04**	1.4 ± 0.1	0.6 ± 0.2**	1.1 ± 0.1	1.2 ± 0.03*	0.9 ± 0.09	1.1 ± 0.04
24 h	2.3 ± 0.05**	3.4 ± 0.3**	1.8 ± 0.09**	1.7 ± 0.04**	2.1 ± 0.07**	1.8 ± 0.4	1.3 ± 0.04*
48 h	1.6 ± 0.07**	2.6 ± 0.2**	1.0 ± 0.1	1.5 ± 0.01*	2.2 ± 0.08**	1.1 ± 0.05	1.2 ± 0.05

The expression of *HMGCR, ApoE, ABCA1, NPC1, NPC2, LXRα*, and *LXRβ *was examined in GaMg cells exposed to clozapine (25 μM), olanzapine (10 μM), haloperidol (10 μM) or imipramine (10 μM) for 6, 12, 24 and 48 hours. The relative level of gene expression was measured by Q-RT-PCR and normalised against vehicle exposed culture at each timepoint. The data represent mean ± SEM of four independent replicates. * indicates p < 0.05 and ** indicates p < 0.01 as compared to vehicle-exposed cells at the given time.

**Figure 1:**
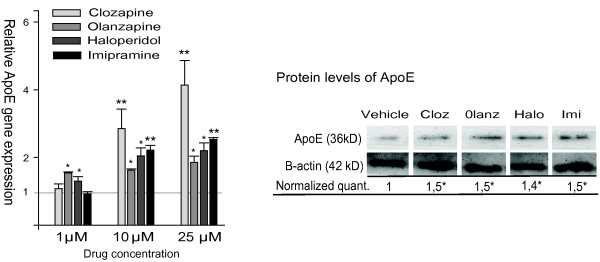
**The relative gene expression and protein levels of ApoE in psychotropic drug-exposed human glioma (GaMg) cells**. Left panel: The relative level of *ApoE *mRNA, measured by Q-RT-PCR after 24 hours exposure, of 1 μM, 10 μM or 25 μM of drug as compared to vehicle-exposed controls. The bars represent mean values ± SEM (n = 3). Right panel: The protein level of ApoE determined by western blotting in GaMg cells exposed to 25 μM of the drug (clozapine, olanzapine, haloperidol and imipramine) or vehicle for 24 hours. The data are normalized relative to the level of beta-actin, showing mean values ± SEM (n = 3) of the ratio between the drug- and vehicle-exposed cells. * indicates p < 0.05, ** indicates p < 0.01.

As shown in Figure 1, the drug effect on *ApoE *expression was increased markedly from 1 μM to 10 μM, and clozapine was apparently the most potent drug at the highest concentration (25 μM). We also examined the protein level of ApoE in GaMg cells exposed to a high dose (25 μM) of clozapine, olanzapine, haloperidol or imipramine for 24 hours. All drugs induced a statistically significant but moderate (1.4-1.5-fold) elevation of ApoE, as compared to the control (Figure 1, right panel). We observed a similar moderate increase in the protein level of NPC1 (about 1.4-fold, data not shown).

**Figure 2:**
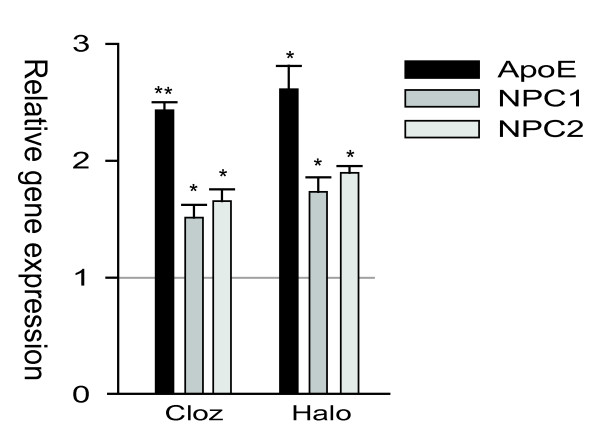
**The relative expression levels of ApoE, NPC1 and NPC2 in psychotropic drug-exposed cultured human astrocytoma (CFF-STTG1) cells**. CFF-STGG1 cells were exposed to 25 μM of clozapine or haloperidol for 24 hours. The data represents mean values ± SEM (n = 3). * indicates p < 0.05, ** indicates p < 0.01.

To investigate whether the psychotropic drug-mediated transcriptional activation of cholesterol transport genes was specific for the GaMg cells, we exposed two other CNS cell lines to clozapine and haloperidol. In cultured human astrocytoma cells (CCF-STTG1), both haloperidol and clozapine significantly up-regulated *ApoE, NPC1 *and *NPC2 *at 24 hours of incubation (n = 3; Figure 2). The maximal response was recorded for the expression of *ApoE*, which reached a 2.4- (*p = 0.001*) and 2.6-fold (*p = 0.008*) increase in the mRNA level after exposure to clozapine (25 μM) and haloperidol (25 μM), respectively. This drug-induced effect on cholesterol transport/export was also observed in cultured human neuroblastoma cells (SH-SY5Y), with clozapine (25 μM) and haloperidol (25 μM) enhancing the expression of *ApoE *about 1.9-fold (*p = 0.007*) and 2.5-fold (*p = 0.001*) at 24 hours, respectively. In contrast, there was apparently no change in the *NPC1 *mRNA level, and the *NPC2 *gene was not expressed in quantifiable amounts in the SH-SY5Y cells (data not shown).

**Figure 3:**
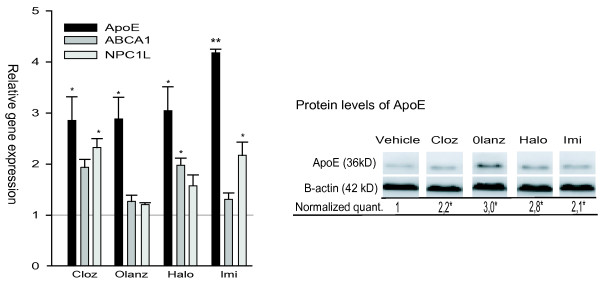
**The relative expression levels of ApoE, ABCA1, NPC1L1 and protein levels of ApoE in psychotropic drug-exposed cultured human hepatoma (HepG2) cells**. Left panel: Gene expression levels in HepG2 cells were exposed to 25 μM of clozapine, olanzapine, haloperidol or imipramine, for 24 hours. The data represents mean values ± SEM (n = 4). Right panel: The protein level of ApoE determined by western blotting in HepG2 cells exposed to 25 μM of the drug (clozapine, olanzapine, haloperidol and imipramine) or vehicle for 24 hours. The data are normalized relative to the level of beta-actin, showing mean values ± SEM (n = 3) of the ratio between the drug- and vehicle-exposed cells. * indicates p < 0.05, ** indicates p < 0.01.

The liver is a major site for peripheral control of lipid homeostasis, and we therefore also examined the expression of cholesterol transport genes in cultured human HepG2 hepatoma cells. At 25 μM, clozapine, olanzapine, haloperidol and imipramine all mediated about 3-fold up-regulation of *ApoE *gene expression, with imipramine as the most potent drug (Figure 3). In addition, *ABCA1 *gene expression was significantly up-regulated in both the clozapine- and haloperidol exposed cells. HepG2 is one of few cell lines expressing the NPC-related protein NPC1L1. We found a significant 2-fold up-regulation of the *NPC1L1 *gene expression by clozapine- and imipramine exposure (Figure 3). The protein level of ApoE was also significantly increased (2.1-3.0-fold, *p < 0.05*) for all drugs at 25 μM concentration, as shown in Figure 3, right panel.

## Discussion

In this study, we have shown how three antipsychotic drugs (clozapine, olanzapine and haloperidol) and one tricyclic antidepressant drug (imipramine) increase the expression of central genes involved in cellular cholesterol transport and export. A transcriptional response was observed in all human cell lines (GaMg glioblastoma, CCF-STTG1 astrocytoma, SH-SY5Y neuroblastoma and HepG2 hepacytoma cells) examined, although with various selections of drugs and concentrations and with some notable differences. The up-regulation of the LXR target genes *ApoE, NPC1 *and *NPC2 *was not evident before 24 to 48 hours of drug exposure, being clearly delayed as compared to the activation of SREBP-controlled lipid biosynthesis, thereby indicating that the increased expression of cholesterol transport genes is secondary to the SREBP-mediated response. Also, the increase in LXR transcripts was modest. This observation is in line with data showing that the LXR transcription factor can be activated by *de novo *synthesized oxysterols [19]. The drug-induced increase in gene expression also produced a moderate elevation of intracellular ApoE protein levels in GaMg and HepG2 cells.

Changes in apolipoprotein E and D levels in the CNS have previously been reported in patients with schizophrenia and bipolar disorder [20,21]. The psychotropic drug-induced activation of ApoE reported here is compatible with clinical data showing increased ApoE levels after treatment with mood-stabilizing and antipsychotic drugs (although only a trend for the latter case), whereas untreated patients with schizophrenia spectrum- and bipolar disorder have decreased levels [22]. Our findings may in part explain the mechanism behind the observed changes in ApoE levels.

Involvement of myelin- and oligodendrocyte (glial) abnormalities is suggested to play an important role in the etiology of schizophrenia, bipolar disorder and unipolar depression [8,10,23,24]. ApoE together with cholesterol is essential in generation and maintenance of myelin [5] and these factors have also been demonstrated to comprise a rate-limiting glia-derived growth factor important in the formation of synapses and dendrite maturation in cultured neuronal cells [6,25]. A recent study found that polymorphisms in the SREBPF1 and SREBF2 genes are associated with schizophrenia, suggesting that variation in lipid biosynthesis affects disease susceptibility [26]. These genes encode the SREBP transcription factors that control the biosynthesis of cholesterol and fatty acids. Due to restricted transport of lipoproteins across the blood-brain barrier, a complete machinery of both cholesterol production and transport is needed to maintain cholesterol homeostasis in the CNS [5]. We found that LXR-controlled intracellular cholesterol transport proteins investigated were up-regulated by the psychotropic drugs. ABCA1 is primarily involved in the formation of HDL-particles and this process is a rate-limiting step in cellular cholesterol efflux and reverse cholesterol transport [27]. Interestingly, clozapine and haloperidol induced an up- and down-regulation of *ABCA1 *expression in GaMg-cells respectively, but this divergent action was not observed in HepG2-cells. Enhancement of *ABCA1 *expression requires a potent activation of both LXR and SREBP [28], whereas an imbalanced stimulation of these transcription factors may lead to down-regulation of *ABCA1 *expression [29,30]. A transcriptional response with up-regulation of many lipid transport genes, except for *ABCA1*, is similar to the expression profile seen in Niemann Pick type C1-knock-out mice, leading to perturbed myelin structure and neurological symptoms due to accumulation of cholesterol in lysosomes/endosomes [31]. Treatment with LXR-agonists and induction of *ABCA1 *in these NPC1 knock-out animals reduced neuroinflammation, attenuated neurodegeneration, and prolonged their lifespan [31]. Recent studies show that some atypical antipsychotic drugs as well as the antidepressant drug imipramine affect myelination [9,32,33] and neuroplasticity [34], but the underlying molecular mechanisms remain unknown. Although care should be taken when extrapolating data from cultured cancer cells to *in vivo *situation, the effect of psychotropic drugs on lipid biosynthesis along with elevated ApoE expression and cholesterol transport could represent an important psychopharmacological effect of these drugs.

Many psychotropic agents have high propensity to induce metabolic adverse effects, such as weight gain and dyslipidemia, which represent major a clinical problem in the treatment of psychiatric patients [35,36]. In a clinical setting, we have demonstrated that, in line with our initial findings in cultured cells, the expression of SREBP-controlled fatty-acid biosynthesis genes *(FASN *and *SCD*) is increased in blood cells of olanzapine-treated patients as compared to unmedicated controls [15]. In addition, a variant of the *INSIG2 *gene in the SREBP-system, was found to be associated with antipsychotic drug-induced weight gain [37]. Activation of SREBP- and LXR-transcription factors can alter body cholesterol homeostasis, not only through production and export, but also via changes in intestinal cholesterol absorption. The *Niemann-Pick type C1-like 1 (NPC1L1) *gene, which was up-regulated in the HepG2 hepatoma cells by several psychotropic drugs, is essential for intestinal cholesterol absorption [38]. Our present data raise the possibility that the drug-mediated effects on LXR-pathways are involved in the molecular mechanisms of drug-induced weight gain and dyslipidemia, since LXR-controlled genes influence lipid transport from peripheral tissues to the bloodstream. Interestingly, an association has been shown between clinical effect and weight gain in clozapine-treated patients [39] and another study suggested that increased serum lipid levels predicts the clinical response to clozapine treatment [40].

## Conclusion

Our data show as a proof-of-principle that psychotropic drug-induced stimulation of cellular lipid biosynthesis *in vitro *is followed by a transcriptional activation of cholesterol transport and -efflux, including ApoE. Such drug-effects could influence myelin maintenance, synaptogenesis and drug-induced metabolic disturbances. Since our data are obtained in cultured cells, further research is needed to explore if these processes are also present *in vivo*.

## Methods

### Cell cultures and drug exposure

Four human cell lines were used in this study: glioblastoma- (GaMg), astrocytoma- (CCF-STTG1), neuroblastoma- (SH-SY5Y) and hepatocellular carcinoma (HepG2) cells. GaMg was obtained from an in-house source and cultured as previously described [2]. CCF-STTG1 (catalog no. CRL-1718), SH-SY5Y (catalog no. CRL-2266) and HepG2 (catalog no. HB-8065) were purchased from ATCC and cultured according to the manufacturer's recommendations . All cells were grown in monolayer in 6-well plates (TPP supplied by Medprobe, Oslo, Norway) in 5% CO_2 _at 37°C for 24 hours before adding fresh medium with drug or vehicle. Cell cultures were exposed to haloperidol (Sigma Aldrich, St Louis, USA), clozapine (Sigma Aldrich, St Louis, USA), olanzapine (Toronto Research Chemicals, Toronto, Canada), imipramine (ICN Biomedicals, Irvine, USA) or vehicle (6 μg/ml lactic acid) equal to solvent.

### Gene expression

Total RNA was extracted using the ABI PRISM™ 6100 Nucleic Acid PrepStation (Applied Biosystems, Foster City, USA), quality-controlled by Agilent Bioanalyzer 2100 (Agilent Technologies, Palo Alto, USA) and quantified using Nanodrop ND-1000 spectrophotometer (Nanodrop Technologies, Delaware, USA). All RNA samples were stored at -80°C until use. cDNA synthesis and quantitative real-time PCR (Q-RT-PCR) was performed with Taqman Gold kit from Applied Biosystems and run on the ABI PRISM 7900HT sequence detector as described previously [2]. Primers (Sigma Genosys, Haverhill, UK) used in this study: *HMGCR *(NM_000859): FWD 5'-tgaagctttgccctttttcctac-3' REV 5'-attttcccttacttcatcctgtgag-3'; *ApoE *(NM_000041): FWD 5'-agctcccaggtcacccag-3' REV 5'-caccggggtcagttgttcc-3'; *ABCA1 *(NM_005502): FWD 5'-tccaggccagtacggaattc-3' REV 5'-actttcctcgccaaaccagtag-3'; *LXRα *(NM_005693): FWD 5'-aagccctgcatgcctacgt-3' REV 5'-gtgggaacatcagtcggtcat-3'; *LXRβ *(NM_007121): FWD 5'-gagggagcagtgcgtcctt-3' REV 5'-gctgttgtttccgaatcttcttc-3'. *NPC1, NPC2 *and *NPC1L1 *primers were purchased as Assay on Demand (Applied Biosystems Inc, Foster City, USA), assay number Hs00264835_m1 (Nm_002713), Hs00197565_m1 (Nm_006432.5) and Hs00203602_m1 (Nm_001101648.1 and Nm_013389.2), respectively.

### Immunoblot analysis

For western blot experiments, the cell culture medium was supplemented to contain 20% FBS for three hours prior to addition of the drugs or vehicle, in order to reduce the basal level of SREBP-controlled lipid biosynthesis as described previously [1,3]. Collection of total cell protein was performed by washing the adherent cells twice with cold PBS before adding RIPA lysis buffer (containing 15 mM NaCl, 50 mM TRIS, 0.5% sodium deoxycholate, 1% Np-40 and 0.1% SDS) with protease inhibitor (Roche Diagnostics, Indianapolis, USA). The cell lysates were standardized according to their protein content as determined by the Bio-Rad technique (Bio-Rad Laboratories, Richmond, USA) and subjected to SDS-PAGE using NuPage gels and nitrocellulose membranes on the Western Breeze system (Invitrogen, New York, USA), according to the manufacturer's recommendations. The membranes were incubated for three hours with purified monoclonal anti-human mouse antibody against β-actin (Nordic Biosite, Täby, Sweden), washed twice and then incubated overnight at 4°C with purified monoclonal anti-human mouse antibodies against ApoE (BD Biosciences Pharmingen, San Diego, USA) or anti-human mouse antibodies against NPC1 (Zymed, San Francisco, USA). The blots were probed with a common secondary antibody solution, followed by detection using Chemiluminescence reagent (Invitrogen, New York, USA) according to the manufacturer's instructions. Quantification of western blots was performed by densitometry and image scanning with Fuji Las-1000 luminescent image analyzer (Fuji Film Co, Fuji, Japan) and Image Gauge v4.0 software (Fuji).

### Statistical analysis

All quantitative real-time PCR data were tested using one-way ANOVA with Dunnett's 2-sided post-hoc test in SPSS 14.0. Two-sided student t-test with unequal variances assumed was used to test the statistical significance of the western blot quantification. The threshold for statistical significance was set at *p = 0.05*.

## Authors' contributions

AOVM conceived the study, performed the experiments, analyzed data and drafted the manuscript. JF, SS and VMS co-conceived the study, supervised the work and co-authored the manuscript. All authors have read and approved the final manuscript.

## References

[B1] Raeder MB, Ferno J, Vik-Mo AO, Steen VM (2006). SREBP activation by antipsychotic- and antidepressant-drugs in cultured human liver cells: relevance for metabolic side-effects?. Mol Cell Biochem.

[B2] Ferno J, Skrede S, Vik-Mo AO, Havik B, Steen VM (2006). Drug-induced activation of SREBP-controlled lipogenic gene expression in CNS-related cell lines: marked differences between various antipsychotic drugs. BMC Neurosci.

[B3] Ferno J, Raeder MB, Vik-Mo AO, Skrede S, Glambek M, Tronstad KJ, Breilid H, Lovlie R, Berge RK, Stansberg C (2005). Antipsychotic drugs activate SREBP-regulated expression of lipid biosynthetic genes in cultured human glioma cells: a novel mechanism of action?. Pharmacogenomics J.

[B4] Raeder MB, Ferno J, Glambek M, Stansberg C, Steen VM (2006). Antidepressant drugs activate SREBP and up-regulate cholesterol and fatty acid biosynthesis in human glial cells. Neurosci Lett.

[B5] Dietschy JM, Turley SD (2004). Thematic review series: brain Lipids. Cholesterol metabolism in the central nervous system during early development and in the mature animal. J Lipid Res.

[B6] Mauch DH, Nagler K, Schumacher S, Goritz C, Muller EC, Otto A, Pfrieger FW (2001). CNS synaptogenesis promoted by glia-derived cholesterol. Science.

[B7] Thomas EA, Dean B, Pavey G, Sutcliffe JG (2001). Increased CNS levels of apolipoprotein D in schizophrenic and bipolar subjects: implications for the pathophysiology of psychiatric disorders. Proc Natl Acad Sci USA.

[B8] Hakak Y, Walker JR, Li C, Wong WH, Davis KL, Buxbaum JD, Haroutunian V, Fienberg AA (2001). Genome-wide expression analysis reveals dysregulation of myelination-related genes in chronic schizophrenia. Proc Natl Acad Sci USA.

[B9] Garver DL, Holcomb JA, Christensen JD (2008). Compromised myelin integrity during psychosis with repair during remission in drug-responding schizophrenia. Int J Neuropsychopharmacol.

[B10] Tkachev D, Mimmack ML, Ryan MM, Wayland M, Freeman T, Jones PB, Starkey M, Webster MJ, Yolken RH, Bahn S (2003). Oligodendrocyte dysfunction in schizophrenia and bipolar disorder. Lancet.

[B11] Huang TL, Chen JF (2005). Serum lipid profiles and schizophrenia: effects of conventional or atypical antipsychotic drugs in Taiwan. Schizophr Res.

[B12] Vestri HS, Maianu L, Moellering DR, Garvey WT (2007). Atypical antipsychotic drugs directly impair insulin action in adipocytes: effects on glucose transport, lipogenesis, and antilipolysis. Neuropsychopharmacology.

[B13] Yang LH, Chen TM, Yu ST, Chen YH (2007). Olanzapine induces SREBP-1-related adipogenesis in 3T3-L1 cells. Pharmacol Res.

[B14] Minet-Ringuet J, Even PC, Valet P, Carpene C, Visentin V, Prevot D, Daviaud D, Quignard-Boulange A, Tome D, de Beaurepaire R (2007). Alterations of lipid metabolism and gene expression in rat adipocytes during chronic olanzapine treatment. Mol Psychiatry.

[B15] Vik-Mo AO, Birkenaes AB, Ferno J, Jonsdottir H, Andreassen OA, Steen VM (2008). Increased expression of lipid biosynthesis genes in peripheral blood cells of olanzapine-treated patients. Int J Neuropsychopharmacol.

[B16] Adams CM, Goldstein JL, Brown MS (2003). Cholesterol-induced conformational change in SCAP enhanced by Insig proteins and mimicked by cationic amphiphiles. Proc Natl Acad Sci USA.

[B17] Ulven SM, Dalen KT, Gustafsson JA, Nebb HI (2005). LXR is crucial in lipid metabolism. Prostaglandins Leukot Essent Fatty Acids.

[B18] Vance JE, Karten B, Hayashi H (2006). Lipid dynamics in neurons. Biochem Soc Trans.

[B19] Repa JJ, Mangelsdorf DJ (2002). The liver X receptor gene team: potential new players in atherosclerosis. Nat Med.

[B20] Dean B, Laws SM, Hone E, Taddei K, Scarr E, Thomas EA, Harper C, McClean C, Masters C, Lautenschlager N (2003). Increased levels of apolipoprotein E in the frontal cortex of subjects with schizophrenia. Biol Psychiatry.

[B21] Digney A, Keriakous D, Scarr E, Thomas E, Dean B (2005). Differential changes in apolipoprotein E in schizophrenia and bipolar I disorder. Biol Psychiatry.

[B22] Dean B, Digney A, Sundram S, Thomas E, Scarr E (2008). Plasma apolipoprotein E is decreased in schizophrenia spectrum and bipolar disorder. Psychiatry Res.

[B23] Davis KL, Haroutunian V (2003). Global expression-profiling studies and oligodendrocyte dysfunction in schizophrenia and bipolar disorder. Lancet.

[B24] Rajkowska G, Miguel-Hidalgo JJ (2007). Gliogenesis and glial pathology in depression. CNS Neurol Disord Drug Targets.

[B25] Goritz C, Mauch DH, Nagler K, Pfrieger FW (2002). Role of glia-derived cholesterol in synaptogenesis: new revelations in the synapse-glia affair. J Physiol Paris.

[B26] Le Hellard S, Mühleisen TW, Djurovic S, Ferno J, Ouriaghi Z, Mattheisen M, Vasilescu C, Raeder MB, Hansen T, Strohmaier J (2008). Polymorphisms in SREBF1 and SREBF2, two antipsychotic-activated transcriptions factors controlling cellular lipogenesis, are associated with schizophrenia in German and Scandinavian samples. Mol Psychiatry.

[B27] Boadu E, Francis GA (2006). The role of vesicular transport in ABCA1-dependent lipid efflux and its connection with NPC pathways. J Mol Med.

[B28] Tamehiro N, Shigemoto-Mogami Y, Kakeya T, Okuhira K, Suzuki K, Sato R, Nagao T, Nishimaki-Mogami T (2007). Sterol regulatory element-binding protein-2- and liver X receptor-driven dual promoter regulation of hepatic ABC transporter A1 gene expression: mechanism underlying the unique response to cellular cholesterol status. J Biol Chem.

[B29] Zeng L, Liao H, Liu Y, Lee TS, Zhu M, Wang X, Stemerman MB, Zhu Y, Shyy JY (2004). Sterol-responsive element-binding protein (SREBP) 2 down-regulates ATP-binding cassette transporter A1 in vascular endothelial cells: a novel role of SREBP in regulating cholesterol metabolism. J Biol Chem.

[B30] Engelking LJ, Kuriyama H, Hammer RE, Horton JD, Brown MS, Goldstein JL, Liang G (2004). Overexpression of Insig-1 in the livers of transgenic mice inhibits SREBP processing and reduces insulin-stimulated lipogenesis. J Clin Invest.

[B31] Repa JJ, Li H, Frank-Cannon TC, Valasek MA, Turley SD, Tansey MG, Dietschy JM (2007). Liver X receptor activation enhances cholesterol loss from the brain, decreases neuroinflammation, and increases survival of the NPC1 mouse. J Neurosci.

[B32] Xiao L, Xu H, Zhang Y, Wei Z, He J, Jiang W, Li X, Dyck LE, Devon RM, Deng Y (2008). Quetiapine facilitates oligodendrocyte development and prevents mice from myelin breakdown and behavioral changes. Mol Psychiatry.

[B33] Chen F, Madsen TM, Wegener G, Nyengaard JR (2008). Changes in rat hippocampal CA1 synapses following imipramine treatment. Hippocampus.

[B34] Bartzokis G, Lu PH, Nuechterlein KH, Gitlin M, Doi C, Edwards N, Lieu C, Altshuler LL, Mintz J (2007). Differential effects of typical and atypical antipsychotics on brain myelination in schizophrenia. Schizophr Res.

[B35] ADA, Association AP, Endocrinologists AAoC, Obesity NAAftSo (2004). Consensus development conference on antipsychotic drugs and obesity and diabetes. Diabetes Care.

[B36] Nasrallah HA (2006). Metabolic findings from the CATIE trial and their relation to tolerability. CNS spectrums.

[B37] Le Hellard S, Theisen FM, Haberhausen M, Raeder MB, Ferno J, Gebhardt S, Hinney A, Remschmidt H, Krieg JC, Mehler-Wex C (2008). Association between the insulin-induced gene 2 (INSIG2) and weight gain in a German sample of antipsychotic-treated schizophrenic patients: perturbation of SREBP-controlled lipogenesis in drug-related metabolic adverse effects?. Mol Psychiatry.

[B38] Altmann SW, Davis HR, Zhu LJ, Yao X, Hoos LM, Tetzloff G, Iyer SP, Maguire M, Golovko A, Zeng M (2004). Niemann-Pick C1 Like 1 protein is critical for intestinal cholesterol absorption. Science.

[B39] Bai YM, Lin CC, Chen JY, Lin CY, Su TP, Chou P (2006). Association of initial antipsychotic response to clozapine and long-term weight gain. Am J Psychiatry.

[B40] Procyshyn RM, Wasan KM, Thornton AE, Barr AM, Chen EY, Pomarol-Clotet E, Stip E, Williams R, Macewan GW, Birmingham CL (2007). Changes in serum lipids, independent of weight, are associated with changes in symptoms during long-term clozapine treatment. J Psychiatry Neurosci.

